# Plug Closure for Ascending Aortic Pseudoaneurysms After TAVI Using Self-Expandable Valve

**DOI:** 10.1016/j.atssr.2024.04.026

**Published:** 2024-05-23

**Authors:** Yoshiaki Katada, Yoshihito Irie, Yoshito Yamamoto, Hitoshi Nakanowatari, Yoshiki Endo, Akinobu Kitagawa, Yasuhisa Fukada

**Affiliations:** 1 Department of Cardiovascular Surgery, Iwaki City Medical Center, Iwaki, Japan; 2 Department of Radiology, Tokyo Medical University Ibaraki Medical Center, Ibaraki, Japan; 3 Department of Cardiology, Iwaki City Medical Center, Iwaki, Japan

## Abstract

A 90-year-old man received a diagnosis of ascending aortic pseudoaneurysms after transcatheter aortic valve implantation (TAVI) using an Evolut PRO valve (Medtronic). Plug closure of the pseudoaneurysms was successfully performed, and the symptoms improved after the procedure. However, on postoperative day 4, the patient experienced sudden massive hemoptysis and died. Although there are a few reports of successful plug closure of ascending aortic pseudoaneurysms after TAVI, it may be difficult to achieve control of pseudoaneurysms by plug closure alone.

Transcatheter aortic valve implantation (TAVI) is widely used for patients who are unsuitable candidates for open surgical aortic valve replacement (AVR). Ascending aortic pseudoaneurysms after TAVI are reported as very rare complications,^1^^,^^2^ and they often have a fatal course. We report a case of plug closure of ascending aortic pseudoaneurysms that developed 2 years after TAVI using an Evolut PRO valve (Medtronic). However, after the plug closure the patient experienced massive hemoptysis that proved fatal.

A 90-year-old man with a history of TAVI using a 26-mm Evolut PRO valve 24 months earlier presented to Iwaki City Medical Center emergency department with a report of anterior chest discomfort. Follow-up computed tomography (CT) at 12 months after TAVI had shown no abnormal findings, but contrast-enhanced CT during the emergency admission demonstrated 2 pseudoaneurysms in the ascending aorta and a hematoma in the mediastinum (Figure 1). The patient was urgently hospitalized, and laboratory data revealed a serum C-reactive protein level of 15.5 mg/dL. A preliminary blood culture reported a positive result for *Streptococcus constellatus*, but the final report had negative results. Echocardiography did not reveal any vegetations, and the modified Duke criteria were negative for endocarditis. After admission, the patient had several fever spikes and mild hemoptysis. The serum C-reactive protein level gradually decreased with antibiotic administration.

**Figure 1:**
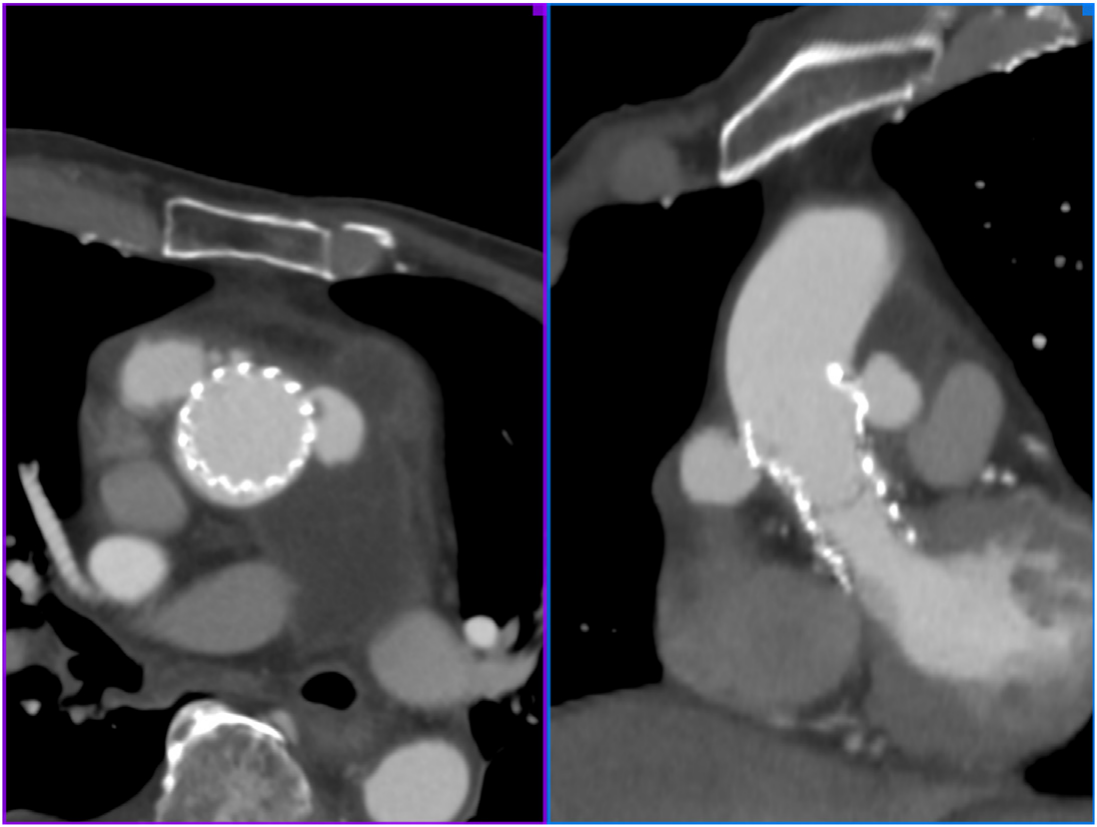
Multiplanar reconstruction views of computed tomography on admission. Two pseudoaneurysms are visualized on both sides of the ascending aorta near the distal stent site, and a massive hematoma is also visualized around the pseudoaneurysm.

The operative mortality/morbidity risk was calculated as 13.1%/46.7% from the Society of Thoracic Surgeons score and as 10.7%/28.7% from JapanSCORE2. We judged that device explantation, surgical AVR, and ascending aortic replacement were unsuitable options. However, because the patient had a low score on the frailty scale and was independent in daily living, we decided to perform urgent plug closure rather than just observe the course.

Plug closure was performed while the patient was under general anesthesia. The orifice of the left pseudoaneurysm appeared considerably larger than on preoperative CT and formed a huge ulcer. To cover the ulcer of the pseudoaneurysm as much as possible, a guiding sheath was advanced into the pseudoaneurysm through the right brachial artery, and a 22-mm Amplatzer vascular plug 2 (AVP2; Abbott Vascular) was placed to cover the orifice of this pseudoaneurysm. Part of the ulcer wall of pseudoaneurysm could not be covered, but a high degree of stagnation of blood flow was achieved at the base of the pseudoaneurysm (Figure 2A). Next, a guiding sheath was advanced through the femoral artery into the pseudoaneurysm formed on the lateral side, and a 16-mm AVP2 was deployed. The third disc of the AVP2 was placed between the aortic wall and the stent strut (Figure 2B). Final aortography showed almost complete disappearance of blood flow into the pseudoaneurysms, although the uncovered ulcer wall remained (Figure 2C). CT confirmed that both AVP2s were firmly implanted and had not migrated.

**Figure 2:**
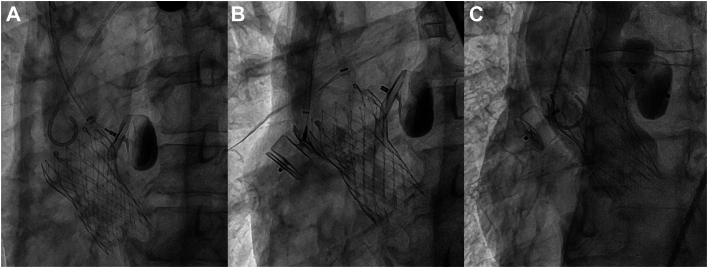
(A) Part of the ulcer wall could not be covered, but complete stoppage of the blood flow at the bottom was achieved. (B) The third disc of the plug was placed between the aortic wall and the stent strut. (C) Completion angiogram. The blood flow into the pseudoaneurysm on the right side has completely disappeared.

After plug closure, the patient reported improvement in his anterior chest discomfort, although he continued to have mild hemoptysis. However, on postoperative day 4, he experienced sudden massive hemoptysis and died.

## Comment

Ascending aortic pseudoaneurysm after TAVI is considered a very rare occurrence, whereas prosthetic valve endocarditis after TAVI has been reported in 3.25% of patients.^3^ In the Surgical EXPLANTation After TAVR Failure (EXPLANT-TAVR) study,^4^ conducted to evaluate cases of explantation after TAVI device implantation, endocarditis was the indication in 43.1% of cases, but ascending aortic pseudoaneurysm was the indication in only a very few cases.

There have been no reports of a large number of cases of ascending aortic pseudoaneurysms after TAVI because of the low incidence rate. However, there are several reports of surgical explantation performed in patients with or without endocarditis,^1^^,^^5^ as well as some reports of plug closure of pseudoaneurysms in the absence of endocarditis at the edge of the self-expanding device,^2^ The EXPLANT-TAVR study reported that, in addition to AVR or aortic root replacement, ascending aortic replacement was performed in 12.4% of cases, with an operative mortality of 0.7% and in-hospital mortality of 11.9%^4^; these findings indicate that the operative risk is still high. Furthermore, although not reported in the literature, the operative and in-hospital mortality could be expected to be even higher when the mortality analysis is limited to cases of ascending aortic pseudoaneurysms.

Although no studies including a large number of cases of plug closure have been reported, plug closure is expected to be applicable to many patients because of the relatively low invasiveness of the procedure. However, in the case of self-expanding types of device series, it is necessary to approach the pseudoaneurysm through the stent strut of the device, and robust plug closure may be difficult. There are some reports of plug closure through the device strut,^2^ which requires complete closure of the entry sites with the disc located in the pseudoaneurysm.

We approached the pseudoaneurysms from the superior margin of the stent, and when the third disc was deployed, it was possible to deploy it between the stent strut and the aortic wall. In cases of surgical explantation, adhesions to the prosthetic valve are often observed around the aortic valve, whereas adhesions to the aortic wall are often absent in the flaring portion of the stent of the self-expanding device. To ensure reliable plug closure, it is necessary to deploy the third disc between the stent strut and the aortic wall whenever possible. However, in our case, the pseudoaneurysm on the medial side had a different morphology compared with the preoperative CT, and it is highly likely that intimal inflammation was still active at the plug closure. The failure to cover the entire intimal defect area with a plug could have allowed intimal destruction to continue after closure in the areas that were not covered by the plug, thus resulting in early postoperative rupture of the pseudoaneurysm.

There have been few reports of plugging of ascending aortic pseudoaneurysms after TAVI, and almost all cases have been reported only with short-term outcomes.^2^^,^^6^ Long-term outcomes are still unclear. In our case, the patient died of massive hemoptysis on postoperative day 4, and there is no disputing that surgical repair is still the gold standard for treating ascending aortic pseudoaneurysms after TAVI. However, some patients, such as very old and frail patients, are not suitable candidates for surgical repair, and the endo-Bentall procedure^7^ may be necessary in such cases.
